# Spinal versus General Anaesthesia in Postoperative Pain Management during Transurethral Procedures

**DOI:** 10.5402/2011/895874

**Published:** 2011-07-12

**Authors:** Stavros I. Tyritzis, Konstantinos G. Stravodimos, Ioanna Vasileiou, Georgia Fotopoulou, Georgios Koritsiadis, Vasileios Migdalis, Anastasios Michalakis, Constantinos A. Constantinides

**Affiliations:** ^1^Department of Urology, Athens University Medical School, LAIKO Hospital, Athens 11527, Greece; ^2^Department of Anesthesiology, LAIKO Hospital, Athens 11527, Greece; ^3^Department of Urology, 251 Airforce General Hospital, Athens 11527, Greece

## Abstract

We compared the analgesic efficacy of spinal and general anaesthesia following transurethral procedures. 97 and 47 patients underwent transurethral bladder tumour resection (TUR-B) and transurethral prostatectomy (TUR-P), respectively. Postoperative pain was recorded using an 11-point visual analogue scale (VAS). VAS score was greatest at discharge from recovery room for general anaesthesia (*P* = 0.027). The pattern changed significantly at 8 h and 12 h for general anaesthesia's efficacy (*P* = 0.017 and *P* = 0.007, resp.). A higher VAS score was observed in pT2 patients. Patients with resected tumour volume >10 cm^3^ exhibited a VAS score >3 at 8 h and 24 h (*P* = 0.050, *P* = 0.036, resp.). Multifocality of bladder tumours induced more pain overall. It seems that spinal anaesthesia is more effective during the first 2 postoperative hours, while general prevails at later stages and at larger traumatic surfaces. Finally, we incidentally found that tumour stage plays a significant role in postoperative pain, a point that requires further verification.

## 1. Introduction

Pain management during transurethral procedures is a major concern. Apart from the standard general anaesthesia, regional anaesthesia is also extensively applied. Nevertheless, the administered type of anaesthesia is ultimately based on the anaesthesiologist's decision. Regional anaesthesia is divided in epidural, spinal, and saddle blockade anaesthesia. Recently, local anesthesia with infiltration of the bladder wall or periprostatic nerve blockage was reported [1–3]. Additionally, sedoanalgesia which is the combination of local anaesthesia with sedation or even the use of virtual reality for pain management has been examined [4, 5]. 

The selection of anaesthesia in a transurethral prostatectomy (TUR-P) or a transurethral bladder tumor resection (TUR-B) has been investigated meticulously in previous reports [6–8]. In general, regional or local anaesthesia demonstrates distinct advantages and disadvantages in terms of postoperative morbidity [6–8]. However, to our knowledge, there is no study comparing these methods. The aim of the present study was to compare general and spinal anaesthesia in terms of postoperative pain mitigation by recording the patient's pain perception during the critical first 24 postoperative hours and to investigate a potential correlation of clinical and pathological data with the pain induced by the transurethral procedures.

## 2. Patients and Methods

**Figure 1:**
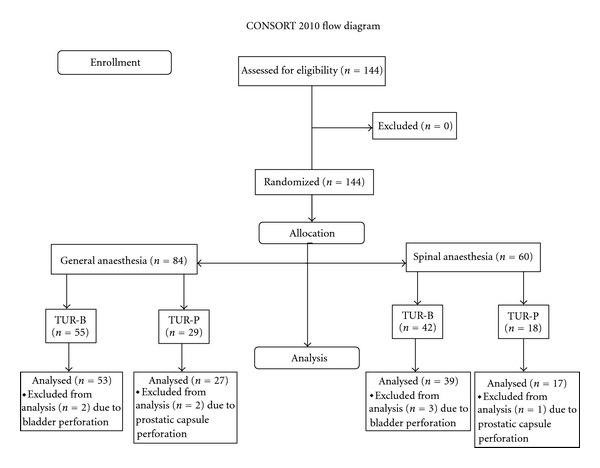
Randomization of the patients according to the CONSORT flow diagram.

All patients provided informed consent, before being included in the study. Distribution of the patients is depicted in the CONSORT flow diagram [9] (Figure 1). Patient age and distribution of the population according to stage and grade of the transitional cell carcinomas (TCC) are summarized in Table 1. 

Conventional transurethral procedures were performed using standard resectoscopes and electrocautery. No preemptive analgesia was administered. Induction to general anaesthesia was done with the intravenous (I.V.) administration of 1-2 g/kg of propofol, followed by 1-2 mg/kg of fentanyl along with 1-2 mg/kg of suxamethonium. Maintenance of anaesthesia was achieved with the I.V. administration of 0.5 mg/kg of atracurium (N_2_O at 50% O_2_) and inhaled desflurane at MAC 1 (Minimum Alveolar Concentration). Spinal anaesthesia was administered as a single shot of 2 mL bupicaine and 1 mL lidocaine without adrenaline. Patients with bladder or prostatic capsule perforation, which was identified intraoperatively, were excluded from the study. A 22-F, 3-way Dufour catheter was placed in all patients, and bladder irrigation was standard. Bladder irrigation was stopped after the completion of the 24-hour observation period after verifying the absence of intravesical clotting by manual irrigation and suction. Patients needing further irrigation were also excluded.

Postoperative pain severity was assessed and recorded using an 11-point visual analogue scale (VAS). The VAS was given to each patient at the time of his/her arrival in the recovery room, which was considered as hour 0. Each patient recorded his/her pain tolerance at postoperative hours (h) 0, 2, 4, 8, 12, and 24. No pain scored 0 points, while worst possible pain for the patient was scored with 10 points. The pain scoring was reviewed by an anesthesiologist.

In cases with VAS scale score > 3 during the observation period, 500 mg of paracetamol combined with 20 mg of hyoskine N-butylbromide (Buscopan) were administered. 

Stratification of pain scoring was made using several parameters, such as age, gender, stage, grade, and location of the TCC's.

Statistical analysis was performed using the SPSS 14.0 for Windows statistical package (SPSS, Inc., Chicago, Il, USA). Clinical and demographical characteristics were compared using the Mann-Whitney *U* test for continues variables and the chi-square test for categorical variables. Odds ratios were used to quantify the strength of association between variables. Kruskal-Wallis test was used to estimate equality of population medians among groups and the Mann-Whitney *U* test for comparison between the groups. The Spearman correlation coefficient (when appropriate) was used to examine the independence between categorical variables. Results were considered significant if *P* < 0.05.

## 3. Results

**Figure 2:**
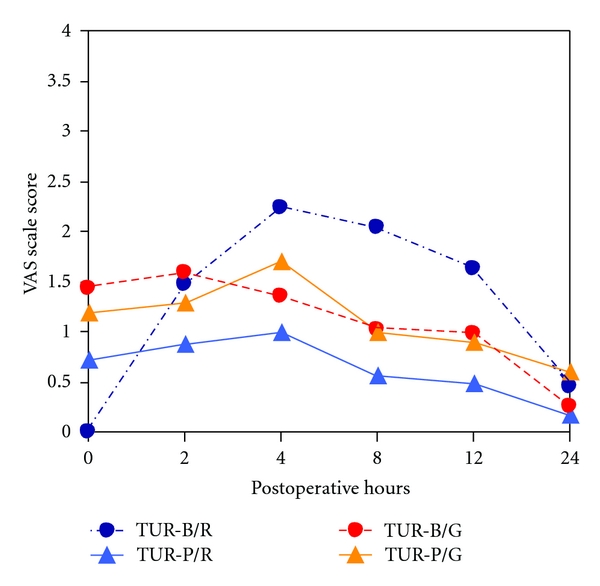
VAS scale score from 0 h to 24 h in TUR-B and TUR-P patients stratified by the type of anesthesia (TUR-B: transurethral bladder tumor resection, TUR-P: transurethral prostatectomy, R: regional anaesthesia, G: general anaesthesia).

VAS scores, with 95% confidence interval (CI) in relation to postoperative time are shown in Figure 2. No statistical difference was detected in the TUR-P patients at any postoperative time between the two anaesthetic methods. In the TUR-B patients, mean (95% CI) analogue VAS score was greatest at 0 h for general versus spinal anaesthesia (*P* = 0.027). At 8 h and 12 h, general anaesthesia's analgesic efficacy was increased significantly (*P* = 0.017 and *P* = 0.007, resp.) 

After adjusting for gender in the TUR-B group, male patients under general anaesthesia experienced more pain at 0 h and 2 h [mean (95% CI), 1 (0–8) versus 0 (0–8) and 1 (0–6) versus 0 (0–10), respectively (*P* = 0.021 and *P* = 0.032)]. However, in the female patients, VAS score was distributed differently, since spinal versus general anaesthesia's analgesic efficacy was lost at 4 h [median (95% CI) 3 (0–4) versus 0 (0–3)], 8 h [2.5 (0–3) versus 0 (0–2)] and 12 h [2 (0–2) versus 0 (0-1)] (*P* = 0.005, *P* = 0.004, *P* < 0.001, resp.), suggesting a better analgesic efficacy for general anaesthesia.

A separate analysis was performed in male patients comparing TUR-P versus TUR-B. No difference in VAS score was recorded between the two surgical approaches when general anaesthesia was chosen as analgesic method. In the spinal group, however, TUR-B patients presented lower mean VAS score than TUR-P patients at 0 h [0 (0–8) versus 0.5 (0–5), (*P* = 0.007)]. This pattern changed at 8 h [2.5 (0–8) versus 0.5 (0–2) (*P* = 0.039)] and at 12 h, [2 (0–8) versus 0 (0–2) (*P* = 0.016)]. 

**Figure 3:**
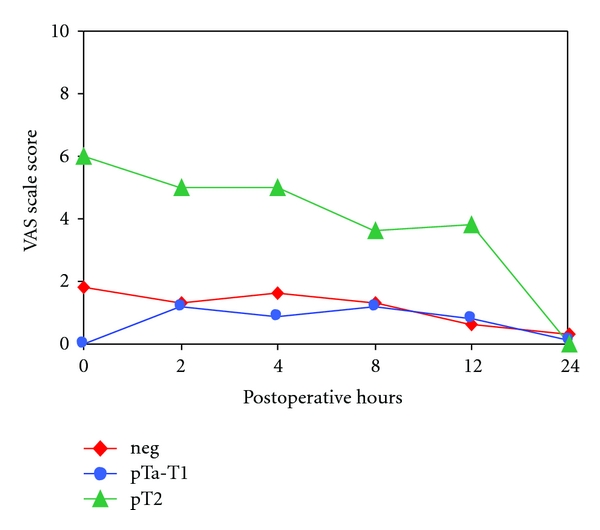
VAS scale score from 0 h to 24 h in TUR-B patients according to tumor stage (Neg: negative pathology report).

Interestingly, after adjusting for the covariates stage and grade in the TUR-B patients, a higher VAS score was observed for pT2 patients compared to pTa, pT1 (TNM), and patients with negative pathology report (Figure 3 and Table 2). Stage and tumor grade were highly correlated (*P* < 0.001), but grade did not present any statistical significance with VAS score at any postoperative hour.

**Figure 4:**
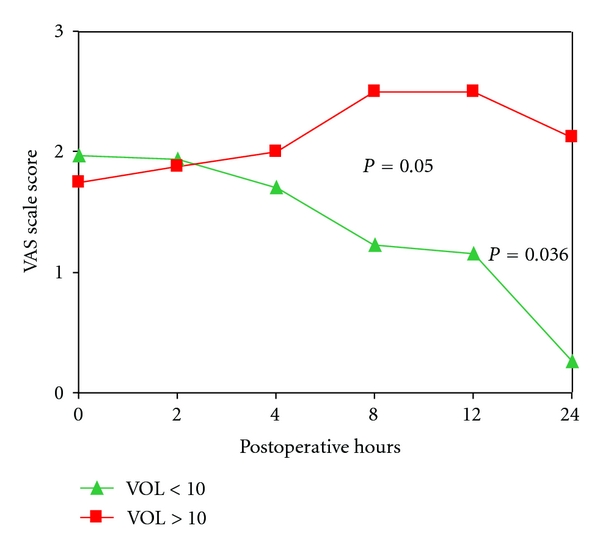
VAS scale score from 0 h to 24 h in TUR-B patients according to resected tumor volume.

Resected tumor volume was used as a categorical variable, and patients were divided in those with <10 cm^3^ [mean volume 3.3 (1–10), (*n* = 82)] and >10 cm^3^ mean 28.1 (11–40) (*n* = 15)]. The cut-off point of 10 cm^3^ was set by the statistical analysis. Those with resected tumor volume > 10 cm^3^ presented VAS score >2 at 8h and 24 h, which was statistically significant (*P* = 0.050, *P* = 0.036, resp.) (Figure 4). Adjusting for the method of anaesthesia, subjects with bladder tumors larger than 10 cm^3^ that received general anaesthesia presented VAS score <3 at 4 h, (OR = 6 95% CI 1.01–35.04, *P* = 0.035). However at 24 h, VAS > 3 was more common in patients with tumor volume <10 cm^3^ who underwent general anaesthesia (OR = 3.5 95% CI 1.09–11.02, *P* = 0.008). 

Finally, location of the bladder tumor was also examined. Apart from the 24 h, tumor located in the lateral bladder walls induced more pain than those situated in the trigone. Moreover, tumor multifocality had the highest VAS scores in all observation periods (Table 3).

## 4. Discussion

The majority of published reports investigate the results of general and spinal anaesthesia separately. Several papers compare the effect of these methods in terms of peri- and postoperative morbidity (blood loss, side effects, and possible complications) [10]. To our knowledge, this is one of the few attempts of comparing the 2 methods by recording the patient's pain perception and tolerance.

Several interesting and novel points were drawn from the statistical analysis of our data. Firstly, in patients undergoing TUR-P, we found that none of the 2 methods of anaesthesia prevailed during the 24-hour observation period. Reeves and Myles reported a similar result using a 5-point verbal rating scale. This result however, described the satisfaction with analgesia and not the pain level [11]. Another report by Fredman and colleagues advocated general anaesthesia as the method of choice in transurethral procedures [12]. Again, patient satisfaction was recorded, but the main limitation was that no adjustment for the type of the transurethral procedure was made. Nott et al. suggested that regional anaesthesia reduces the incidence of catheter-related pain, although being of similar efficacy to oral diazepam and thus is more advantageous than general anaesthesia [13]. The same effect is produced by periprostatic nerve blockage [14]. 

On the other hand, differences were recorded in TUR-B patients. To be more specific, spinal was more efficient than general anaesthesia in the first 2 h after surgery, while general proved to be better in the later time points. This fact can be explained by the major implications induced in bladder function by regional anesthesia. Indeed, catheter-related pain and detrusor muscle spasm elicited by bladder irrigation can be managed efficiently with regional anaesthesia in the first 2 postoperative hours. However, it simultaneously causes a clinically significant disturbance of bladder function due to interruption of the micturition reflex [15]. Additionally, it has a greater effect on bladder compliance and lowers the intraabdominal pressure [16]. As a result, painful bladder overdistention or even acute retention might occur after the removal of the catheter, due to the long-lasting recovery of the normal bladder function. Based on the clinical experience, the incidence of such an event is rather rare, thus we might implicate the catheter and bladder spasm as the most distressing causes of postoperative pain. However, an interesting analgesic approach could have been the combination of spinal anaesthesia with antimuscarinic pretreatment, such as oxybutnin or tolterodine, or the administration of gabapentin during general anaesthesia, which is shown to significantly reduce catheter-related pain [17, 18].

The above urodynamic effects might be interpreted better in the female patients than in men, since in women there are no prostatic symptoms that could bias the bladder pain, and we should also acknowledge the fact that females perceive pain better than men, as recent reports suggest [19]. In the female population of our study, spinal was less effective than general anaesthesia after the first 4 hours, verifying the possible implications of regional anaesthesia in the female bladder.

A very surprising observation is exhibited in the statistical analysis, concerning the bladder tumor stage. Patients with higher stage experienced a higher level of pain than the ones with localized disease (pTa-pT1). No adequate explanation of this phenomenon is provided by the current literature. One can speculate that infiltrative disease stimulates more nociceptors (highly specialized free endings of sensory nerve fibers) [20]. Even though this is a random result, we believe that it is worth mentioning, since it exhibited statistical significance, and it might motivate a better anaesthetic approach of patients that present with pT2 disease cystoscopically. It is obvious that verification is required for the analysis of a large cohort of patients, which should be also stratified by the type of the applied anaesthesia.

We also found that the resected bladder tumor volume becomes a significant parameter of pain induction after a TUR-B, when more than 10 cm^3^ are resected. It is rational to believe that a larger traumatic surface elicits more pain. On the other hand, when we adjusted our analysis to the type of anaesthesia, general anaesthesia seemed to be more efficient at 4 h and 24 h. Tumor volume was the only parameter favoring the efficiency of general anaesthesia independently of the postoperative time of observation.

The use of VAS scale for the documentation of pain measurement introduces a study bias per se, since this is a subjective method. To date, no objective recording of pain perception has been described, even though several pain scales assessing the patient's agitation level by recording the facial expression, leg movement, and muscle tension, or even the brain electrophysiologic activity through specialized electroencephalograms (EEG) are presented as promising [21]. Yet, patient self-reporting of pain by verbal rating is considered the gold standard [21]. The use of vital signs, such as heart rate and arterial pressure or even pupil reactions could reflect partially the pain status, but they could be affected also by the postsurgical stress, the patient's comorbidities, or the prescribed medication. Nevertheless, clinically significant differences between VAS scores might be considered realistic anecdotally, when they exceed 4-5 points. Thus, in our study, clinical significant differences were observed in different bladder tumor stages and multiple versus single tumors.

One comment should be also made about pharmacokinetics. The dosage of epidural regimens has an effect of approximately one and a half hours. Therefore, it could not have altered the VAS scoring, since the 0 h point was set at the arrival in the recovery room. The selection of paracetamol and hyoskine N-butylbromide as rescue analgesics in patients, reporting VAS > 3 postoperatively, was made due to their immediate action and their short half-lives [22, 23], which could not have affected the perception of pain during the 2-hour and 4-hour intervals of VAS recording.

This study is not without limitations. The patients were not randomized due to the fact that the selection of analgesia was made by the anaesthesiologists individually for each patient, according to their performance status. Another drawback could be the lack of analysis of the time of the operation and the prostatic volume resected. Even though the resected adenoma cannot be rationally associated with pain induction, since the major causes of TUR-P pain are bladder spasm, catheter-related pain, and capsule offense, a future study could incorporate this parameter. As for the time of the operation, we believe that it could not have affected the result, since the scoring started after the completion of the operation, during which pain is managed sufficiently.

## 5. Conclusions

The analysis of the advantages and disadvantages of regional compared to general anaesthesia is immensely complicated. As can easily be imagined, the secondary effects of both types of anaesthesia are varied, and realistically, they must be considered for each and every patient, due to the unique clinical profiles presented. This study attempted to establish the potency of general and spinal anaesthesia during transurethral procedures by recording the patient's pain perception. It seems that spinal anaesthesia is more effective during the first 2 postoperative hours, while general prevails at later stages and at larger traumatic surfaces. Finally, we incidentally found that tumor stage plays a significant role in postoperative pain, a point that requires further verification. We advocate a closer cooperation of the urologist with the anaesthesiologist in terms of providing more specific information about the patient's disease and tailoring the type of anaesthesia to the patient's needs.

## Figures and Tables

**Table 1:** Clinical characteristics of the patients according to the type of anaesthesia.

	Regional	General	*P*-value
Age (years) (mean ± S.D.)	69.76 ± 7.42	67.22 ± 10.14	NS
Stage (*n*)			
* No cancer*	12	18	
* pTa-pT1*	17	25	NS
* pT2*	13	11	
Grade (*n*)			NS
* No cancer*	12	18	
* GI-GII*	16	16	
* GIII*	14	8	

NS: non significant, M/F: male/female, VAS: visual analogue pain score, median (range) and mean ± standard deviation (S.D.)

**Table 2:** Mean (range) (95% CI) VAS scale score from 0 h to 24 h in TUR-B patients according to tumor stage.

Hours	0	2	4	8	12	24
Negative	1.8 (0–7)	1.3 (0–5)	1.6 (0–6)*	1.3 (0–6)	0.6 (0–4)*	0.3 (0–2)
PTa-T1	0 (0–7)*	1.2 (0–5)*	0.9 (0–5)*	1.2 (0–5)	0.8 (0–8)*	0.1 (0–8)
PT2	6(0–8)^+∗^	5 (0–10)^+∗^	5 (0–10)^+∗^	3.6 (0–8)	3.8 (0–8)^+∗^	0 (0–8)

Kruscall Wallis <0.05, *Mann-Whitney *P* value <0.05 between groups, negative: no urothelial tumor found in specimen.

**Table 3:** Stratification of mean (range) (95% CI) VAS score by bladder tumor location.

	Trigone (*n* = 25)	Lateral wall (*n* = 26)	Multiple tumors (*n* = 18)	*P*-value
VAS at 0 h	0.64* (0.1–1.18)	1.54* (0.5–2.6)	4.06 (2.32–5.79)	0.002
VAS at 2 h	0.56* (0.15–0.97)	1.27* (0.44–2.1)	4.56 (2.69–6.43)	<0.001
VAS at 4 h	0.6* (0.12–1.08)	1.35* (0.55–2.15)	4.44 (2.98–5.9)	<0.001
VAS at 8 h	0.96 (0.37–1.55)	1.08* (0.37–1.55)	3.56 (2.23–4.88)	<0.001
VAS at 12 h	0.68* (0.19–1.17)	0.96* (0.2–1.72)	3.56 (2.02–5.09)	<0.001
VAS at 24 h	0.4* (−0.4–0.12)	0.15* (−0.7–0.37)	2.39 (0.73–4.04)	0.001

*P < 0.05 versus multiple tumors.
